# Characterization of an Aging-Based Diagnostic Gene Signature and Molecular Subtypes With Diverse Immune Infiltrations in Atherosclerosis

**DOI:** 10.3389/fmolb.2021.792540

**Published:** 2022-01-13

**Authors:** Lei Zhao, Fengfeng Lv, Ye Zheng, Liqiu Yan, Xufen Cao

**Affiliations:** Department of Cardiology, Cangzhou Central Hospital, Cangzhou, China

**Keywords:** atherosclerosis, aging-related genes, immune infiltration, diagnosis, molecular subtypes, CEBPB

## Abstract

**Objective:** Advancing age is a major risk factor of atherosclerosis (AS). Nevertheless, the mechanism underlying this phenomenon remains indistinct. Herein, this study conducted a comprehensive analysis of the biological implications of aging-related genes in AS.

**Methods:** Gene expression profiles of AS and non-AS samples were curated from the GEO project. Differential expression analysis was adopted for screening AS-specific aging-related genes. LASSO regression analysis was presented for constructing a diagnostic model, and the discriminatory capacity was evaluated with ROC curves. Through consensus clustering analysis, aging-based molecular subtypes were conducted. Immune levels were estimated based on the expression of HLAs, immune checkpoints, and immune cell infiltrations. Key genes were then identified via WGCNA. The effects of CEBPB knockdown on macrophage polarization were examined with western blotting and ELISA. Furthermore, macrophages were exposed to 100 mg/L ox-LDL for 48 h to induce macrophage foam cells. After silencing CEBPB, markers of cholesterol uptake, esterification and hydrolysis, and efflux were detected with western blotting.

**Results:** This study identified 28 AS-specific aging-related genes. The aging-related gene signature was developed, which could accurately diagnose AS in both the GSE20129 (AUC = 0.898) and GSE43292 (AUC = 0.685) datasets. Based on the expression profiling of AS-specific aging-related genes, two molecular subtypes were clustered, and with diverse immune infiltration features. The molecular subtype–relevant genes were obtained with WGCNA, which were markedly associated with immune activation. Silencing CEBPB triggered anti-inflammatory M2-like polarization and suppressed foam cell formation.

**Conclusion:** Our findings suggest the critical implications of aging-related genes in diagnosing AS and modulating immune infiltrations.

## Introduction

The number of elderly people (aged >65 years) is increasing globally, and cardiovascular diseases, especially atherosclerosis (AS), act as the biggest cause of morbidity and mortality among this population (Lorenzo et al., 2021). AS is the most common underlying pathology of coronary artery diseases, peripheral artery diseases, and cerebrovascular diseases (Wolf and Ley, 2019). It is an aging-related disorder that is in relation to long-term exposure to cardiovascular risk factors. Increasing age acts as an independent risk factor of AS progression (Wang and Bennett, 2012). The aging of the vascular system is a key determinant of the development of AS (de Yébenes et al., 2020). Aging involves cell–cell communications and contributes to distinct stages of AS development (Tyrrell and Goldstein, 2021). In AS lesions, aging may be driven by the exhaustion of replicative potential and a variety of cellular stressors like oxidized LDL (Bian et al., 2020). At present, aging-based therapeutic agents are being sought to treat and prevent AS in humans. Nevertheless, in-depth knowledge is required to fully uncover aging-related genes, to expound aging and AS intertwining, which allows us to develop more effective therapeutic strategies, and to decrease the burden of AS.

AS represents a systemic chronic inflammatory disease of the arterial wall, caused by the accumulation of lipoproteins and the activation of various dysregulated immune cells (Liberale et al., 2020). Aging influences the immune system in complex mechanisms, and diverse components in the immune system contribute to AS progression (Minhas et al., 2021). Immune cell subpopulations of diverse lineages are involved in AS, such as macrophages, dendritic cells, T helper 1 cells, and B cells, all of which may be influenced by aging (Tyrrell and Goldstein, 2021). Some adaptive and innate immune responses are protective, while others trigger the progression of AS (Nilsson and Hansson, 2020). Recently, clinical trials have demonstrated that immune checkpoint inhibitors exhibit the potential of targeting the immune system to combat AS (Ridker et al., 2017). Increasing evidence suggests an inability to fine-control systemic inflammatory responses, which seems to be a sign of failure in aging (Rea et al., 2018). Nevertheless, so far, few studies have been conducted for exploring the interactions of aging-related genes and immune infiltrations in AS. Herein, this study conducted a comprehensive analysis of the biological implications of aging-related genes and their relevance to immune infiltrations in AS.

## Materials and Methods

### Data Acquisition

The mRNA expression profiles of 71 non-AS and 48 AS samples from female peripheral blood were curated from the Gene Expression Omnibus (GEO) repository (https://www.ncbi.nlm.nih.gov/gds/) with the accession number GSE20129, which was used as the training set (Huang et al., 2011). Microarray expression data of 32 pairs of atheroma plaque and control were retrieved from the GSE43292 dataset, as the testing set (Ayari and Bricca, 2013). Transcriptomic profiling and follow-up information of patients who underwent endarterectomy operations were retrieved from the GSE21545 dataset (Jiang et al., 2019; Gallina et al., 2021). The probes were converted to gene symbols in line with the probe annotation files. If more than one probe corresponded to the same gene symbol, the average of these probes was calculated as the expression level of the gene. In total, 307 aging-related genes were downloaded from the Human Ageing Genomic Resources (HAGR; https://genomics.senescence.info/) (Tacutu et al., 2018).

### Differential Expression Analysis

Differential expression analysis of aging-related genes was presented between AS and non-AS samples utilizing the limma package (version 3.40.6) (Ritchie et al., 2015). The Benjamini–Hochberg method was adopted for adjusting the original *p*-values. Genes with the false discovery rate (FDR) < 0.05 were considered AS-specific aging-related genes. The Pearson correlation test was adopted for evaluation of the interactions between AS-specific aging-related genes at the mRNA level.

### Protein–Protein Interaction Analysis

A PPI network was established through the Search Tool for the Retrieval of Interacting Genes/Proteins (STRING; version 11.0) online database (https://string-db.org) (Franceschini et al., 2013). The Molecular Complex Detection (MCODE) plug-in in the Cytoscape software (version 3.7.2) was adopted for screening the significant module, with degree cutoff = 2, K-core = 2, and node score cutoff = 0.2 (Bader and Hogue, 2003).

### Function Enrichment Analysis

Through the clusterProfiler package (version 3.12.0), Gene Ontology (GO) and Kyoto Encyclopedia of Genes and Genomes (KEGG) pathway enrichment analyses of AS-specific aging-related genes were carried out (Yu et al., 2012). Terms with the FDR<0.05 were significantly enriched. Gene Set Enrichment Analysis (GSEA; version 4.1.0) was adopted for identifying the most significant biological processes and KEGG pathways between AS and non-AS groups (Subramanian et al., 2005). “c2.cp.kegg.v7.0.symbols.gmt” derived from the Molecular Signatures Database (MSigDB; version 7.1) was downloaded as the reference gene set (Liberzon et al., 2015).

### Development of an Aging-Related Gene Signature for AS Diagnosis

To minimize the risk of overfitting, the logistic least absolute shrinkage and selection operator (LASSO) regression analysis algorithm was adopted, which used regularization to improve the prediction accuracy (Tibshirani, 1997). Through the glmnet package (Engebretsen and Bohlin, 2019), the genes significantly associated with the discrimination of AS and non-AS specimens were identified. Ten-fold cross-verification was presented for tuning parameter selection. Lambda was determined as the minimum partial likelihood deviance. To verify the predictive significance of the identified aging-related gene signature, receiver operator characteristic (ROC) curves were generated based on the mRNA expression profiles from 71 non-AS and 48 AS patients in the GSE20129 dataset. The area under the ROC curve (AUC) was calculated for determining the diagnostic effectiveness in discriminating AS from non-AS patients and externally verified in the dataset. Moreover, the prognostic value of feature aging-related genes was assessed in the GSE21545 dataset. The cutoff value of each gene was determined, and Kaplan–Meier curves of survival analysis were conducted between groups with the survival package.

### Analysis of Immune Cell Infiltrations and Immunity

The computational single sample gene set enrichment analysis (ssGSEA) method derived from the Gene Set Variation Analysis (GSVA) package (version 1.32.0) was employed for analyzing the immune cell subpopulations across samples from the GSE20129 dataset (Hänzelmann et al., 2013). The enrichment of each immune cell type was scored according to the marker genes of 28 immune cell subpopulations (Charoentong et al., 2017). The mRNA expression of human leukocyte antigen (HLA) molecules and immune checkpoints was quantified in each sample.

### Consensus Clustering Analysis

Consensus clustering analysis was carried out utilizing the ConsensusClusterPlus package (version 1.48.0) on the basis of the mRNA expression profiling of AS-specific aging-related genes (Wilkerson and Hayes, 2010). The Euclidean distance between specimens was determined. To classify AS specimens into diverse subgroups, unsupervised k-means clustering analysis was presented for consensus clustering with 1,000 repetitions based on Euclidean distance. Through the t-distributed stochastic neighbor embedding (t-SNE) method, the difference between clusters was confirmed. Meanwhile, the MSigDB hallmark gene set (h.all.v7.1.symbols.gmt) was adopted for running ssGSEA to reveal the activities of hallmark pathways in two clusters.

### Co-Expression Analysis

The co-expression network was conducted with the weighted gene correlation network analysis (WGCNA) package to uncover the correlation of genes and critical interacted genetic modules based on the mRNA expression profiling of AS-specific aging-related genes (Langfelder and Horvath, 2008). PickSoftThreshold function was utilized for calculating the soft thresholding power β value in line with scale independence and mean connectivity. Co-expression modules with aging-related clusters were then established utilizing blockwiseModules function. Each module was assigned a unique color. The Pearson correlation of each module’s eigengene with phenotypes was analyzed. Afterward, gene significance and module membership indicators were separately calculated by signedKME and cor functions for identifying key genes with gene significance >0.5 and module membership >0.8. Module membership represents the relationships between gene expression profiling and module eigengene. Meanwhile, gene significance represents the absolute value of the associations between gene expression and module traits.

### Cell Culture and Differentiation

Human monocytes THP-1 were maintained in RPMI-1640 medium (Sigma-Aldrich, United States) plus 10% fetal bovine serum (FBS). All monocytes were grown in an environment of 5% CO_2_ at 37°C. THP-1 monocytes were differentiated to macrophages through exposure to 160 nmol/L phorbol 12-myristate 13-acetate (PMA; Sigma-Aldrich, United States) lasting 72 h. THP-1 cells were induced to foamy macrophages by incubation with 100 mg/L ox-LDL lasting 48 h. Macrophages were polarized to M1 macrophages via incubation with 20 ng/ml interferon-γ (IFN-γ) and 10 pg/ml lipopolysaccharide (LPS). Moreover, macrophage M2 polarization was induced by incubating with 20 ng/ml interleukin-4 (IL-4).

### Cell Transfection

The siRNAs against CEBPB (si-CEBPB) and siRNA negative control (si-NC) were retrieved from GenePharma, Inc. (Shanghai, China). Macrophages were transfected with oligonucleotides via Lipofectamine 2000 (Invitrogen, United States) in line with the manufacturer’s instructions. Reverse transcription-quantitative polymerase chain reaction (RT-qPCR) was adopted for evaluating the transfection efficiency of si-CEBPB.

### RT-qPCR

Total RNA was extracted from macrophages utilizing TRIzol reagent (Invitrogen, United States). RNA concentration was determined with Nanodrop 2000. cDNA was synthesized with 500 ng RNA in line with the manufacturer’s instruction. RT-qPCR was presented on a CFX96 Touch Real-Time PCR Detection System (Bio-Rad, United States) utilizing iTaq Universal SYBR Green (Bio-Rad, United States). The primer sequences are listed in Table 1. With GAPDH as a reference control, the expression of target genes was determined with the 2^−ΔΔCt^ method.

**TABLE 1:** Primer sequences of genes by RT-qPCR.

Target genes	Primer sequences
CEBPB	5′-CTT​CAG​CCC​GTA​CCT​GGA​G-3′ (forward)
	5′-GGA​GAG​GAA​GTC​GTG​GTG​C-3′ (reverse)
iNOS	5′-TTC​AGT​ATC​ACA​ACC​TCA​GCA​AG-3′ (forward)
	5′-TGG​ACC​TGC​AAG​TTA​AAA​TCC​C-3′ (reverse)
FIZZ1	5′-CCG​TCC​TCT​TGC​CTC​CTT​C-3′ (forward)
	5′-CTT​TTG​ACA​CTA​GCA​CAC​GAG​A-3′ (reverse)
Ym1	5′-ATC​CAG​TCT​GGC​TAT​GAG​ATC​C-3′ (forward)
	5′-TCA​GTC​GGG​TAT​TTG​TAG​AGG​G-3′ (reverse)
Arg1	5′-GTG​GAA​ACT​TGC​ATG​GAC​AAC-3′ (forward)
	5′-AAT​CCT​GGC​ACA​TCG​GGA​ATC-3′ (reverse)
GAPDH	5′-CTG​GGC​TAC​ACT​GAG​CAC​C-3′ (forward)
	5′-AAG​TGG​TCG​TTG​AGG​GCA​ATG-3′ (reverse)

### Western Blotting

Macrophages were plated in a six-well plate and grown to 60% confluency. After being lysed with RIPA lysis buffer on the ice, 20 μg protein was subjected to SDS-PAGE and transferred onto the polyvinylidene fluoride membrane. Afterward, the membrane was blocked with Tris-buffered saline and Tween 20 (TBST) plus 5% non-fat milk powder for 2 h at room temperature. Then, the membrane was treated with primary antibodies against CEBPB (1:1,000; 23431-1-AP; Proteintech, Wuhan, China), iNOS (1:1,000; 18985-1-AP; Proteintech, Wuhan, China), FIZZ1 (1:1,000; ab271225; Abcam, United States), Ym1 (1:1,000; 21484-1-AP; Proteintech, Wuhan, China), Arg1 (1:5,000; ab233548; Abcam, United States), CD36 (1:1,000; 18836-1-AP; Proteintech, Wuhan, China), LOX-1 (1:1,000; 11837-1-AP; Proteintech, Wuhan, China), SR-A (1:1,000; ab183725; Abcam, United States), SR-B (1:2000; ab206233; Abcam, United States), ABCA1 (1:50,000; ab125064; Abcam, United States), ABCG1 (1:1,000; 13578-1-AP; Proteintech, Wuhan, China), ACAT1, and GAPDH (1:20,000; 60004-1-Ig; Proteintech, Wuhan, China) at 4°C overnight. Afterward, the membrane was washed with TBST three times, followed by incubation with HRP-conjugated goat anti-rabbit or anti-mouse antibody (1:10,000; ab7090 or ab47827; Abcam, United States) at room temperature for 1 h. Immunoreactivity was quantified with an enhanced chemiluminescence detection system. Images were acquired with an automatic digital gel image analysis system.

### Enzyme-Linked Immunosorbent Assay

Levels of inflammatory cytokines including tumor necrosis factor-α (TNF-α), interleukin-6 (IL-6), and IL-1β in culture medium were examined with commercially available ELISA kits (Nanjing Jiancheng Institute of Biological Engineering, China) in line with the manufacturer’s instructions. The absorbance value at 405 nm was tested with a microplate reader.

### Statistical Analysis

Statistical analysis was conducted with GraphPad Prism software (version 8.0.1). All data are displayed as mean ± standard deviation. Comparisons between two groups were evaluated with Wilcoxon’s test or Student’s t-test. Meanwhile, comparisons among three or more groups were carried out with the analysis of variance followed by Tukey’s post hoc test. *p* < 0.05 was set as statistical significance.

## Results

### Expression Patterns and Biological Significance of Aging-Related Genes in AS

**FIGURE 1:**
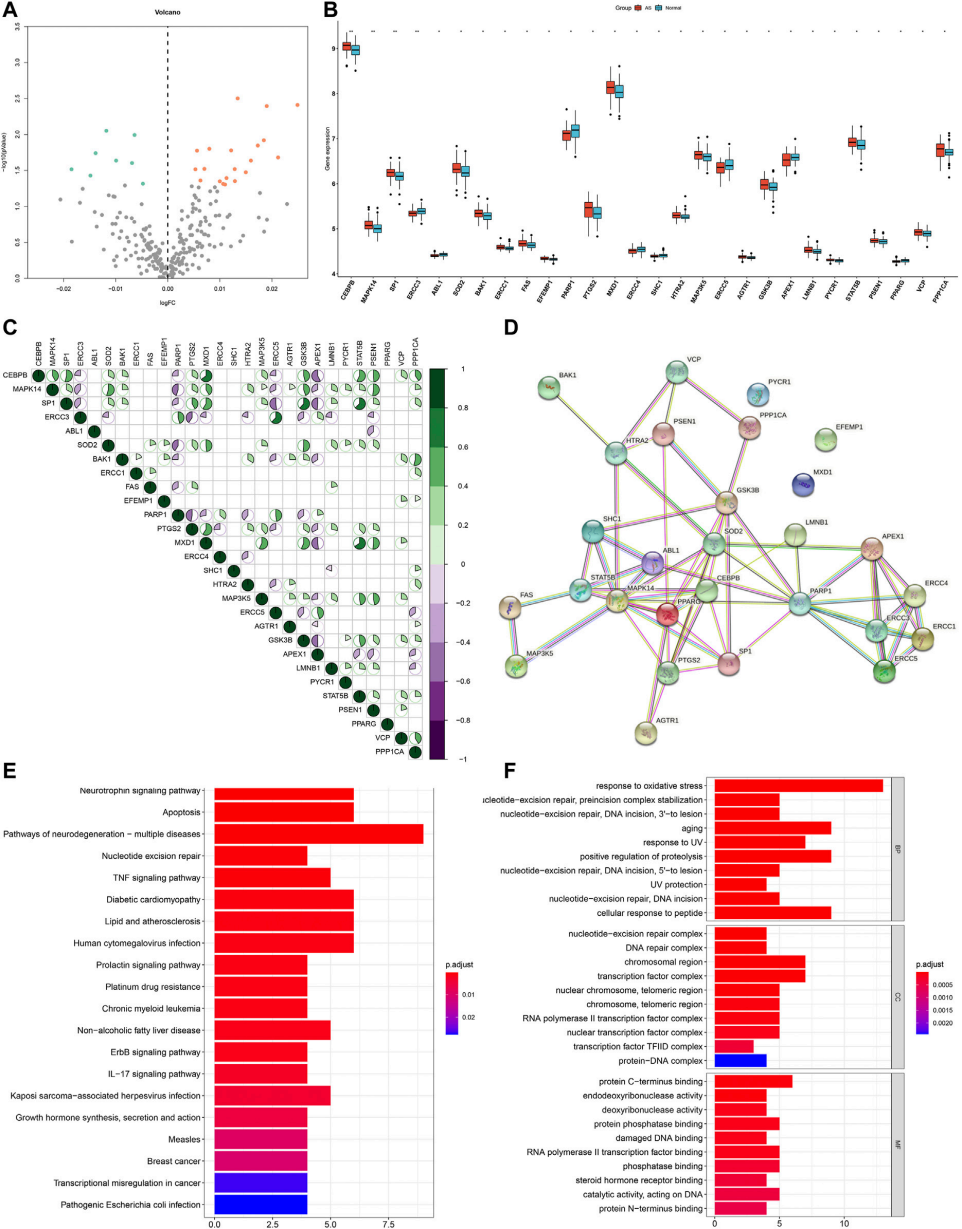
Expression patterns and biological significance of aging-related genes in AS. **(A)** Volcano plots visualizing the expression patterns of aging-related genes in AS and non-AS samples. Orange bubbles mean up-regulated genes, blue bubbles mean down-regulated genes, and gray bubbles mean non-significant genes. **(B)** Box plots showing the differentially expressed aging-related genes in AS and non-AS samples. *p < 0.05; **p < 0.01. **(C)** Pearson correlation between AS-specific aging-related genes among AS samples. Purple represents negative correlation, while green represents positive correlation. **(D)** PPI network of proteins encoded by AS-specific aging-related genes through the STRING database. **(E)** KEGG pathways enriched by AS-specific aging-related genes. **(F)** Biological processes (BPs), cellular components (CCs), and molecular functions (MFs) associated with AS-specific aging-related genes.

This study harvested 307 aging-related genes from the HAGR project. Expression patterns of aging-related genes were analyzed in AS and non-AS samples from the GSE20129 dataset. Our results showed that MAPK14, PTGS2, SP1, SOD2, BAK1, MXD1, GSK3B, CEBPB, STAT5B, MAP3K5, FAS, LMNB1, PPP1CA, VCP, PSEN1, ERCC1, HTRA2, PYCR1, EFEMP1, and AGTR1 expressions displayed marked up-regulation in AS than non-AS samples (Figures 1A,B and Table 2). Meanwhile, PPARG, ABL1, SHC1, ERCC4, ERCC3, PARP1, APEX1, and ERCC5 exhibited more distinct down-regulation in AS than non-AS samples. The above genes were considered AS-specific aging-related genes. Through the Pearson correlation test, there were prominently positive or negative correlations between the AS-specific aging-related genes among AS samples at the mRNA levels (Figure 1C). Moreover, the PPI network uncovered the close interactions between proteins encoded by the AS-specific aging-related genes (Figure 1D). KEGG enrichment results demonstrated that AS-related (lipid and AS) and immunity-related pathways (TNF and IL-17 signaling pathways) were significantly enriched by the AS-specific aging-related genes (Figure 1E). As shown in GO annotation results, the AS-specific aging-related genes were markedly associated with oxidative stress, aging, and DNA repair (Figure 1F).

**TABLE 2:** AS-specific aging-related genes.

Aging-related genes	AS	Non-AS	LogFC	*p*-Value
CEBPB	8.963346	9.047084	0.013416	0.003149
MAPK14	5.015483	5.102793	0.024898	0.003887
SP1	6.157043	6.238663	0.018999	0.004022
ERCC3	5.382679	5.338848	−0.0118	0.008887
ABL1	4.423791	4.404287	−0.00637	0.01016
SOD2	6.251091	6.331509	0.018441	0.012048
BAK1	5.280884	5.344529	0.017283	0.014239
ERCC1	4.56879	4.594123	0.007977	0.015811
FAS	4.642165	4.681445	0.012156	0.016653
EFEMP1	4.323343	4.340194	0.005612	0.016777
PARP1	7.161269	7.092815	−0.01386	0.018185
PTGS2	5.339084	5.418034	0.021177	0.020871
MXD1	8.03037	8.119685	0.015957	0.023058
ERCC4	4.541617	4.510499	−0.00992	0.023063
SHC1	4.41161	4.390684	−0.00686	0.024921
HTRA2	5.272931	5.298685	0.007029	0.030047
MAP3K5	6.583337	6.642216	0.012845	0.03025
ERCC5	6.423041	6.341288	−0.01848	0.030459
AGTR1	4.358364	4.374411	0.005302	0.03046
GSK3B	5.922632	5.984424	0.014974	0.033491
APEX1	6.583896	6.516471	−0.01485	0.037267
LMNB1	4.499552	4.534832	0.011268	0.040335
PYCR1	4.286578	4.305256	0.006273	0.043613
STAT5B	6.86238	6.923944	0.012885	0.044461
PSEN1	4.714857	4.74761	0.009987	0.044754
PPARG	4.289684	4.275532	−0.00477	0.04833
VCP	4.888364	4.924531	0.010635	0.048638
PPP1CA	6.696638	6.747851	0.010991	0.049572

### Development of an Aging-Related Gene Signature for AS Diagnosis

**FIGURE 2:**
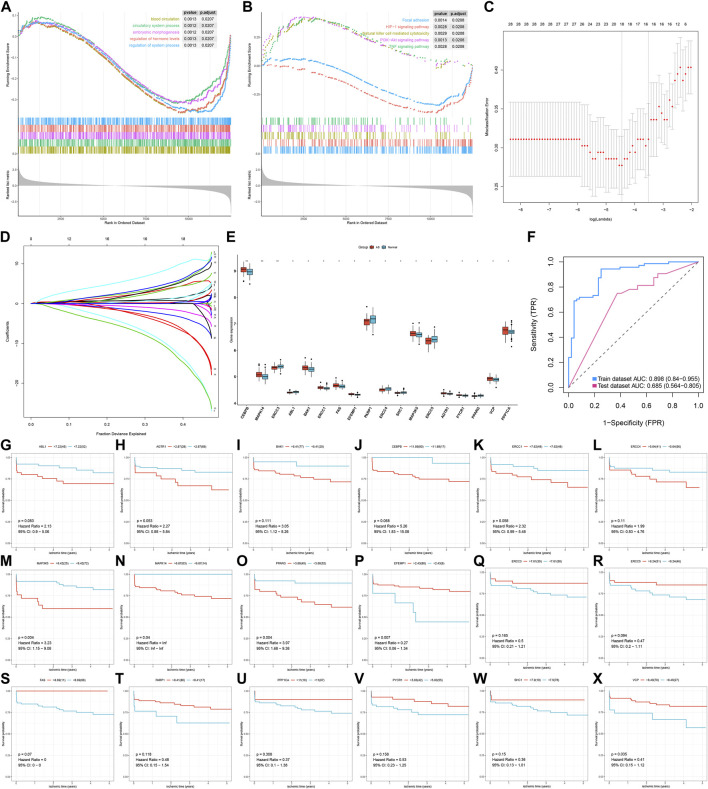
Development of an aging-related gene signature for AS diagnosis. **(A)** GSEA results for the activation of biological processes in AS than non-AS samples. **(B)** GSEA results for the activation of KEGG pathways in AS than non-AS samples. **(C)** Tuning feature selection in the LASSO model. **(D)** LASSO regression coefficient profiles. **(E)** Box plots for the expression of key AS-specific aging-related genes in the LASSO model between AS and non-AS samples. *p < 0.05; **p < 0.01. **(F)** ROC curves for evaluating the diagnostic efficacy of the LASSO model in GSE20129 (training set) and GSE43292 (testing set). **(G–X)** Survival analysis of AS patients with high and low expressions of key AS-specific aging-related genes in the GSE21545 dataset.

Through the GSEA method, we evaluated the differences in activations of biological processes and pathways between AS and non-AS samples. Our results revealed that blood circulation, circulatory system process, embryonic morphogenesis, regulation of hormone levels, and regulation of system process were significantly activated in AS samples (Figure 2A). Meanwhile, focal adhesion, HIF-1 signaling pathway, natural killer cell–mediated cytotoxicity, PI3K–Akt signaling pathway, and TNF signaling pathway exhibited increased activations in AS samples (Figure 2B). This indicated that the above biological processes and pathways could participate in AS progression. Through the LASSO method, feature AS-specific aging-related genes were screened. Based on them, we developed a diagnostic model containing 18 AS-specific aging-related genes (CEBPB, MAPK14, ERCC3, ABL1, BAK1, ERCC1, FAS, EFEMP1, PARP1, ERCC4, SHC1, MAP3K5, ERCC5, AGTR1, PYCR1, PPARG, VCP, and PPP1CA) for AS (Figures 2C,D). Among them, CEBPB, MAPK14, BAK1, ERCC1, FAS, EFEMP1, MAP3K5, AGTR1, PYCR1, VCP, and PPP1CA displayed increased expression in AS compared with non-AS samples (Figure 2E). Meanwhile, ERCC3, ABL1, PARP1, ERCC4, SHC1, ERCC5, and PPARG were markedly reduced in AS than non-AS specimens. The AUC values were separately 0.898 and 0.685 in the GSE20129 and GSE43292 datasets (Figure 2F). This confirmed the excellent diagnostic performance of this model. In the GSE21545 dataset, survival analysis revealed the significant prognostic significance of key AS-specific aging-related genes for AS patients (Figures 2G–X).

### Feature Aging-Related Gene Signatures Associated With Immune Infiltrations in AS

**FIGURE 3:**
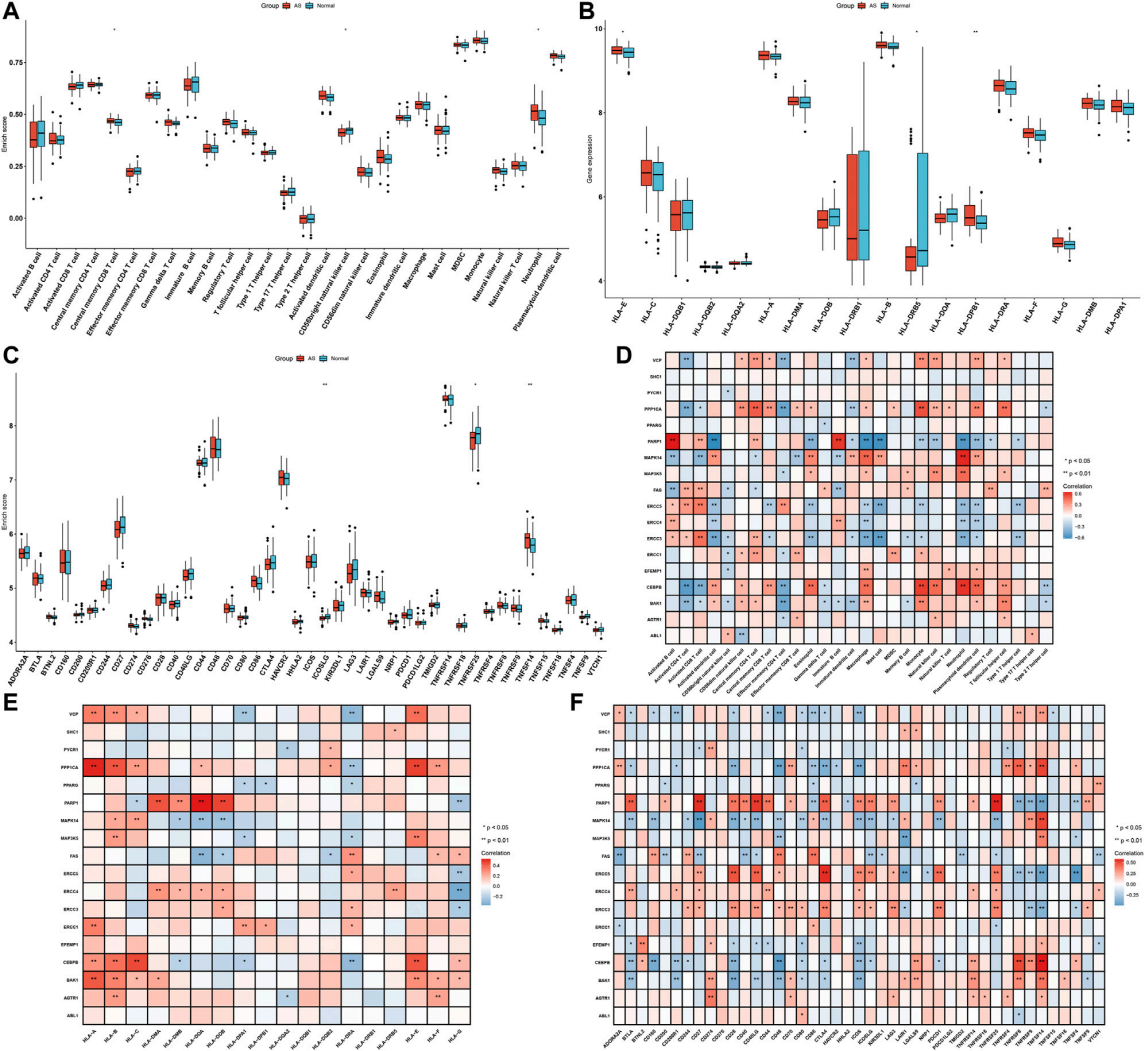
Feature aging-related gene signatures associated with immune infiltrations in AS. **(A)** Box plots showing the differences in immune cell infiltrations between AS and non-AS samples. **(B,C)** Box plots of the mRNA expression of HLA molecules and immune checkpoints in AS and non-AS samples. **(D–F)** Heatmaps visualizing the associations of feature aging-related gene signatures with immune cell infiltrations and mRNA expression of HLA molecules and immune checkpoints across AS samples. *p < 0.05; **p < 0.01.

The differences in immune infiltrations between AS and non-AS samples were evaluated in depth. Compared with those in non-AS samples, we found that the infiltration levels of central memory CD8 T cells and neutrophils were markedly increased in AS samples (Figure 3A). Meanwhile, reduced infiltration levels of CD56bright natural killer cells were observed in AS samples. As shown in Figure 3B, there was significantly increased expression of HLA-E and HLA-DPB1 while reduced expression of HLA-DRB5 in AS than non-AS samples. The expression of immune checkpoints was also compared between AS and non-AS specimens. In comparison with those in non-AS samples, TNFSF14 displayed markedly increased expression, but ICOSLG and TNFRSF25 exhibited prominently reduced expression in AS samples (Figure 3C). The above data indicated the heterogeneity in immune infiltrations between AS and non-AS samples. We further investigated the interactions of feature aging-related gene signatures with immune infiltrations across AS samples. As depicted in Figure 3D, most feature aging-related genes were in relation to immune cell infiltrations. Feature aging-related genes were positively correlated with HLA-A, HLA-B, HLA-DMA, HLA-DRB5, HLA-E, and HLA-F but were negatively associated with HLA-DQA2. Additionally, they were positively or negatively related to the other HLA molecules (Figure 3E). Moreover, there were prominent associations of feature aging-related genes with HLAs and immune checkpoints across AS specimens (Figure 3F).

### Characterization of Two Aging-Related Gene Clusters Across AS Samples

**FIGURE 4:**
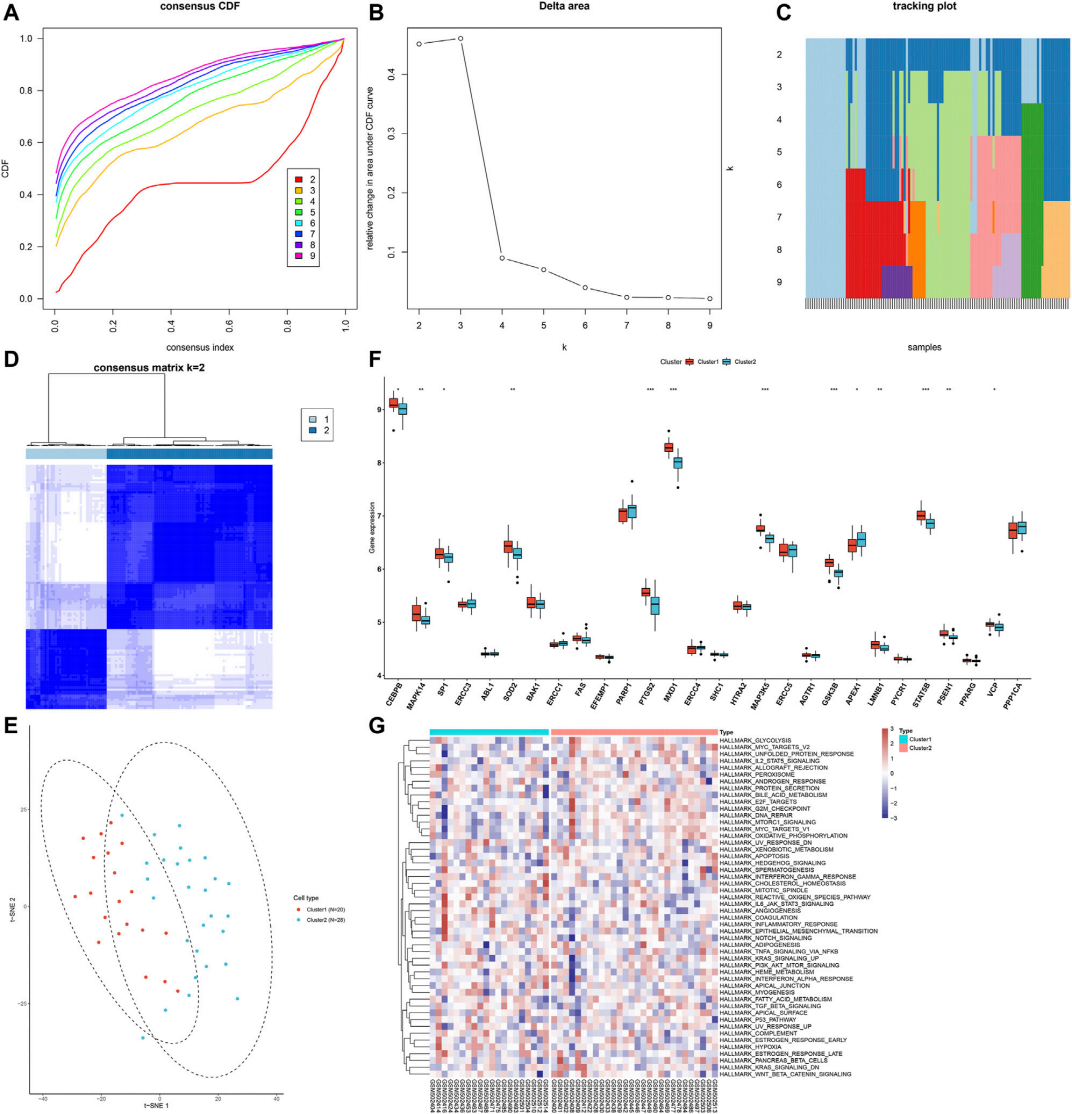
Characterization of two aging-related gene clusters across AS samples. **(A)** Empirical cumulative distribution function (CDF) plots displaying consensus distribution for each k. **(B)** Relative change in the area under the CDF curve. **(C)** Item tracking plot showing the consensus cluster of items (in column) at each k (in row). **(D)** Consensus matrix plots depicting consensus values on a white to blue color scale ordered by consensus clustering when *k* = 2. **(E)** t-SNE plots confirming the classification accuracy of two aging-related gene clusters across AS specimens. **(F)** Box plots showing the difference in mRNA expression of AS-specific aging-related genes between clusters. *p < 0.05; **p < 0.01; ***p < 0.001. **(G)** Heatmap visualizing the activities of hallmark pathways in two aging-related gene clusters.

Through consensus clustering analysis, we clustered AS samples into two molecular subtypes based on the expression profiling of AS-specific aging-related genes, namely, cluster 1 (*n* = 20) and cluster 2 (*n* = 28; Figures 4A–D). The t-SNE plots revealed the prominent differences between samples from the two clusters (Figure 4E). As shown in Figure 4F, the expression of AS-specific aging-related genes displayed the marked heterogeneity between subtypes. We also investigated the activities of hallmark pathways across AS samples. In Figure 4G, reduced activations of glycolysis, MYC targets, unfolded protein response, IL-2–STAT5 signaling, allograft rejection, peroxisome, androgen response, protein secretion, bile acid metabolism, and E2F targets are found in cluster 1 compared with cluster 2.

### Aging-Related Gene Clusters With Different Immune Activations

**FIGURE 5:**
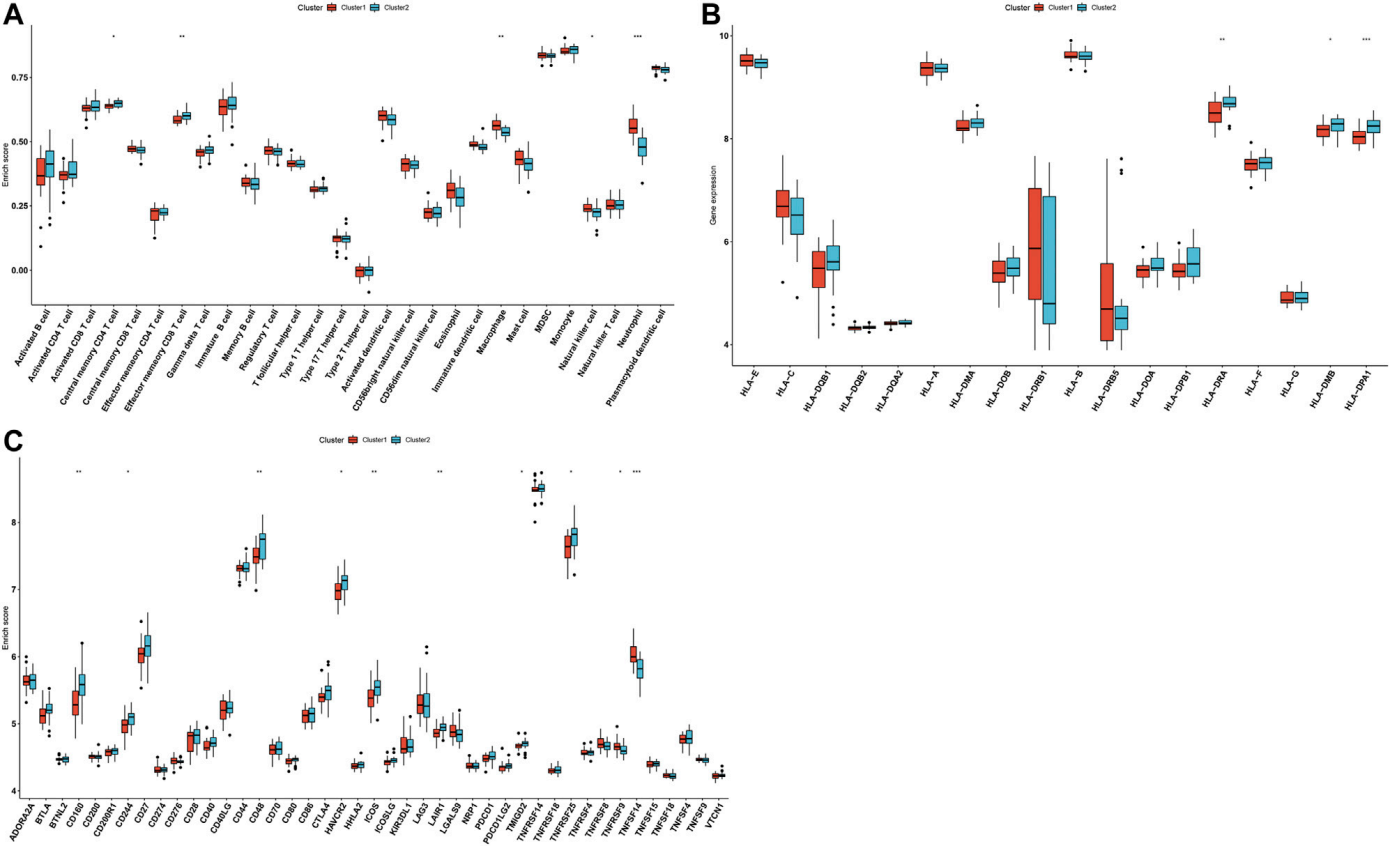
Aging-related gene clusters with different immune activations. **(A)** Box plots of the differences in immune cell infiltrations in aging-related gene clusters 1 and 2. **(B)** Box plots of the differences in mRNA expression of HLA molecules in aging-related gene clusters 1 and 2. **(C)** Box plots of the differences in mRNA expression of immune checkpoints in aging-related gene clusters 1 and 2. *p < 0.05; **p < 0.01; ***p < 0.001.

Through the ssGSEA method, we quantified the infiltration levels of immune cell subpopulations across AS samples from aging-related gene clusters 1 and 2. In Figure 5A, we noticed that compared with those in cluster 1, central memory CD4 T cells and effector memory CD8 T cells exhibited the markedly increased infiltration levels in cluster 2. In contrast, higher infiltration levels of macrophages, natural killer cells, and neutrophils were found in cluster 1 than cluster 2. HLA-DRA, HLA-DMB, and HLA-DPA1 expressions were markedly lowered in cluster 1 than cluster 2 (Figure 5B). Moreover, we found that immune checkpoints including CD160, CD27, CD48, HAVCR2, ICOS, LAIR1, TMIGD2, and TNFRSF25 expressions displayed a marked increase in cluster 2 compared with cluster 1. However, TNFRSF9 and TNFSF14 expressions were prominently higher in cluster 1 than cluster 2 (Figure 5C). Thus, aging-related gene cluster 2 exhibited high immunity activation in AS.

### Establishment of a Co-Expression Network

**FIGURE 6:**
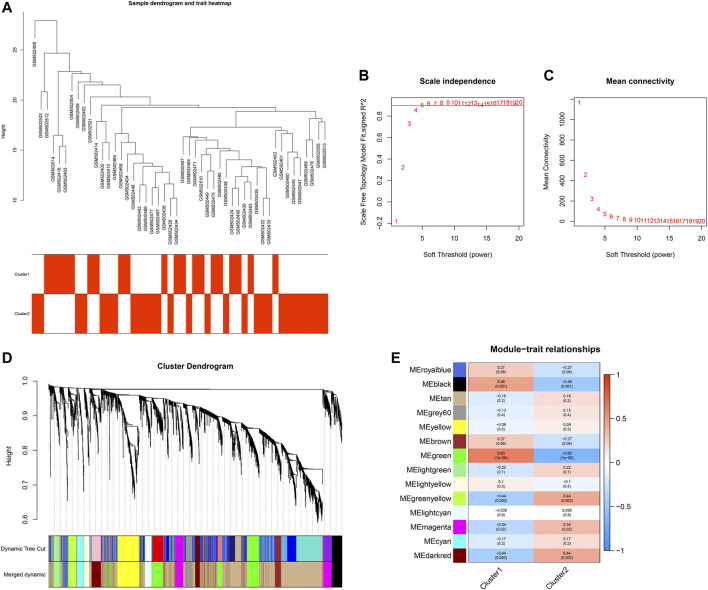
Establishment of a co-expression network. **(A)** Sample dendrogram and trait heatmap. **(B,C)** Scale independence and mean connectivity under diverse soft-thresholding powers. **(D)** Co-expression modules for each gene under the hierarchical clustering tree are assigned diverse colors. The dynamic tree cut corresponds to the original modules, and merged dynamic corresponds to the merged modules finally identified. **(E)** Heatmap of the relationships between co-expression modules and clinical traits. The number indicates the correlation coefficients between co-expression modules and clinical traits, and the number in parentheses indicates the corresponding *p*-values.

Following the quality check of the input data, no specimen was removed (Figure 6A). Here, two phenotypes (clusters 1 and 2) were carried out. In Figures 6B,C, the soft thresholding power is set at 5, while the scale-free topology fit index is up to 0.9, indicative of an approximate scale-free topology. Co-expression modules were established utilizing dynamic tree cut analysis. In total, 14 modules were merged and identified by a unique color (Figure 6D). The gray module contained genes that did not have similar expression patterns and did not belong to any other module. As shown in Figure 6E, the green module displayed the most significant correlation with aging-related clusters, indicating that the genes in the green module were in relation to aging-related clusters.

### Analysis of Genes in Aging-Related Co-Expression Module

**FIGURE 7:**
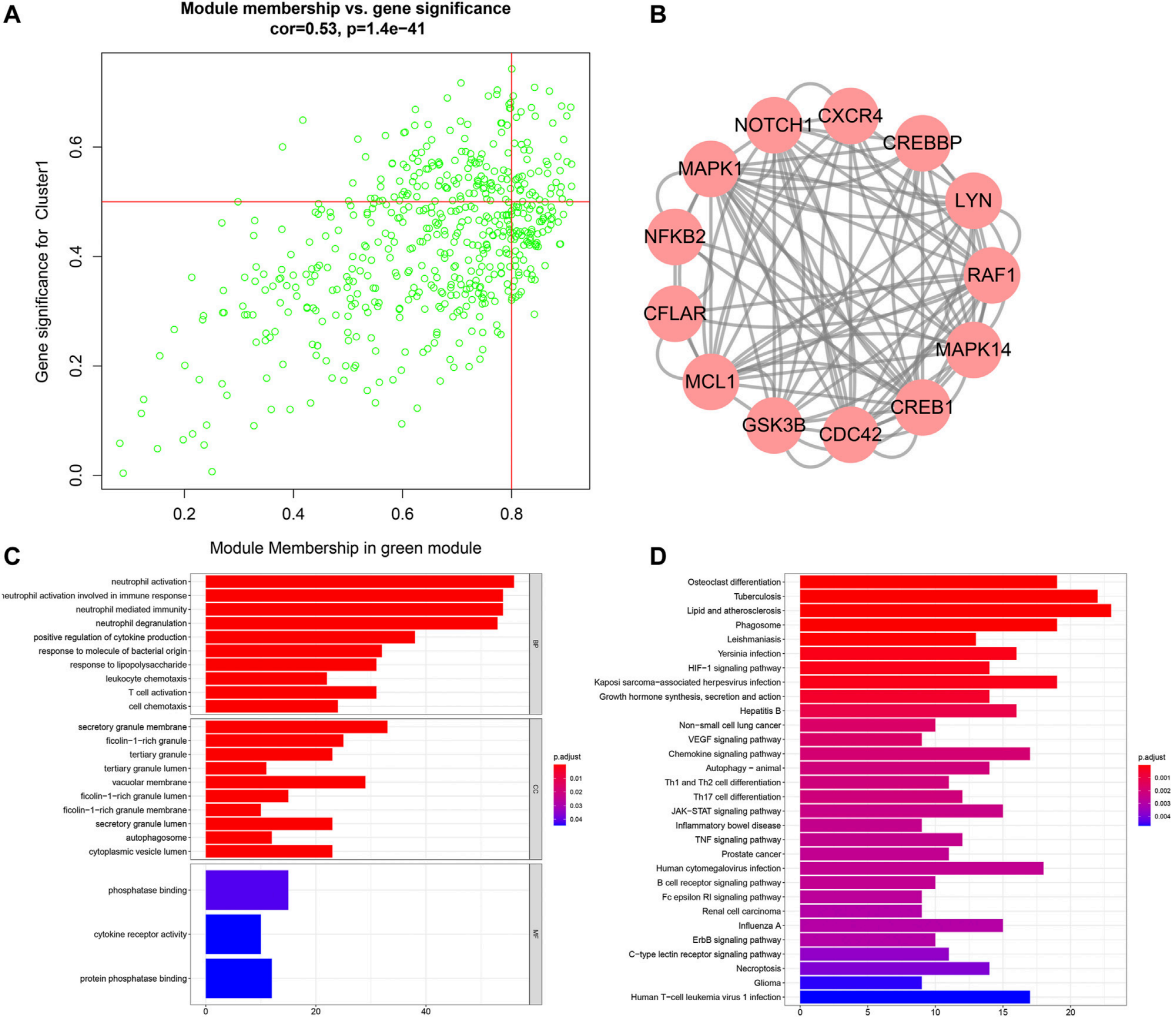
Analysis of genes in the aging-related co-expression module. **(A)** Scatter plots for the interactions between module membership and gene significance for genes in the green module. **(B)** Hub genes among genes in the green module via the MCODE method. **(C)** Biological processes (BPs), cellular components (CCs), and molecular functions (MFs) enriched by genes in the green module. **(D)** KEGG pathways enriched by genes in the green module.

With module membership >0.8 and gene significance >0.5, we identified 49 co-expressed genes highly associated with aging-related clusters in the green module: OSBPL2, FLJ39575, LAT2, TNFRSF10C, KIAA1754, REPS2, DGAT2, C5AR1, NADK, CANT1, BEST1, TIMP2, KIAA0247, LPPR2, IL8RA, ACTN1, ST6GALNAC2, SLC11A1, NCF4, MXD1, MGAM, MEFV, DENND3, TREML2, RAB6IP1, LILRB3, TMCC3, C3orf62, TSEN34, ROPN1L, XPO6, SBNO2, USP32, IL8RB, UBN1, TNFSF14, B4GALT5, CREBBP, MANSC1, PELI2, RAF1, IGF2R, LAMP2, DHX34, DKFZp761E198, ABHD5, RNF24, HAL, and NDEL1 (Figure 7A). Through MCODE, LYN, MCL1, NFKB2, CREB1, CREBBP, NOTCH1, MAPK14, MAPK1, RAF1, CDC42, GSK3B, CXCR4, and CFLAR acted as hub genes (Figure 7B). Function enrichment analysis was presented for uncovering the biological significance of genes in the green module. In Figure 7C, immune-related biological processes are significantly enriched such as neutrophil activation, neutrophil activation involved in immune response, neutrophil-mediated immunity, neutrophil degranulation, positive regulation of cytokine production, leukocyte chemotaxis, and T cell activation. Moreover, we noticed that AS-related (such as lipid and AS, HIF-1 signaling pathway, VEGF signaling pathway, JAK-STAT signaling pathway, TNF signaling pathway, and necroptosis) and immune-related (chemokine signaling pathway, Th1 and Th2 cell differentiation, Th17 cell differentiation, and B cell receptor signaling pathway) pathways were prominently enriched by the genes in the green module (Figure 7D).

### CEBPB Knockdown Triggers Anti-Inflammatory M2-Like Polarization of Macrophages

**FIGURE 8:**
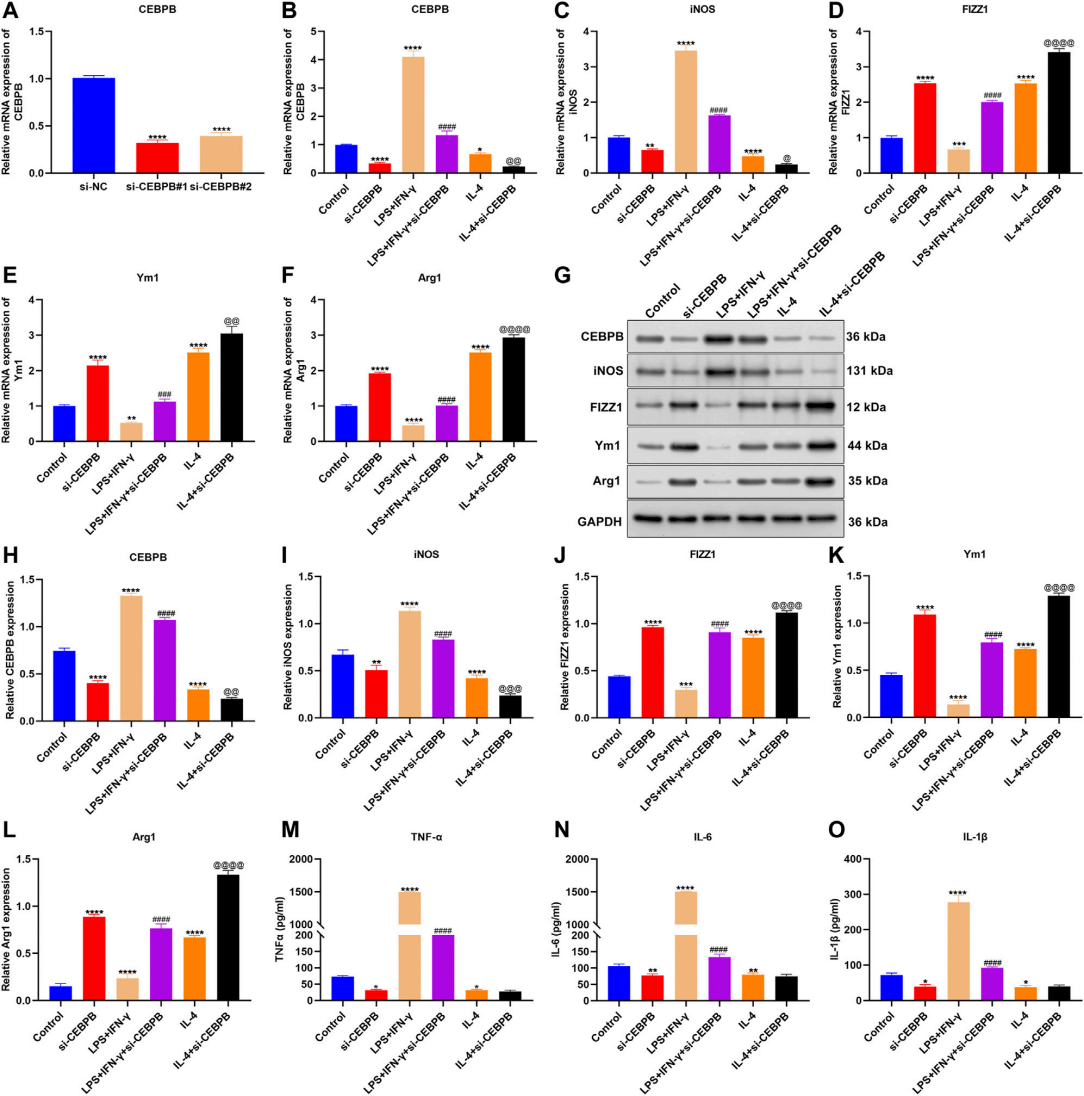
CEBPB knockdown triggers anti-inflammatory M2-like polarization of macrophages. **(A)** RT-qPCR for verifying the expression of CEBPB in macrophages transfected with siRNAs targeting CEBPB. **(B–F)** RT-qPCR for quantifying the expression of CEBPB, M1-type marker (iNOS), and M2-type markers (including FIZZ1, Ym1, and Arg1) in macrophages treated with IFN-γ, and LPS, IL-4, or CEBPB knockdown. **(G–L)** Western blotting for evaluating the expression of CEBPB, M1-type marker (iNOS), and M2-type markers (including FIZZ1, Ym1, and Arg1) in macrophages treated with IFN-γ, and LPS, IL-4, or CEBPB knockdown. **(M–O)** ELISA detecting the levels of TNF-α, IL-6, and IL-1β in the supernatant of macrophages. Compared with control, *p < 0.05; **p < 0.01; ***p < 0.001; ****p < 0.0001. Compared with IFN-γ and LPS, ^###^ *p* < 0.001; ^####^ *p* < 0.0001. Compared with IL-4, ^@^ *p* < 0.05; ^@@^ *p* < 0.01; ^@@@^ *p* < 0.001; ^@@@@^ *p* < 0.0001.

Among AS-specific aging-related genes, CEBPB exhibited marked associations with immune cell infiltrations in AS, especially macrophages. To investigate the function of CEBPB on macrophages, we successfully silenced the expression of CEBPB in macrophages (Figure 8A). Macrophages were exposed to 20 ng/ml IFN-γ and 10 pg/ml LPS to acquire M1 macrophages or incubated with 20 ng/ml IL-4 to acquire M2 macrophages. Increased CEBPB expression was found in M1 macrophages, and its expression was reduced in M2 macrophages (Figure 8B). There were elevated expression of M1-type marker (iNOS) and reduced expression of M2-type markers (including FIZZ1, Ym1, and Arg1) in M1-like macrophages (Figures 8C–F). The opposite results were investigated in M2-like macrophages. Especially, CEBPB knockdown reduced the expression of M1-type marker (iNOS) and enhanced the expression of M2-type markers (including FIZZ1, Ym1, and Arg1). Similar results were observed at the protein levels (Figures 8G–L). These data indicated that CEBPB knockdown promoted M2-like polarization of macrophages. ELISA results demonstrated that silencing CEBPB reduced the LPS-induced and IFN-γ–induced increases in TNF-α, IL-6, and IL-1β in macrophages (Figures 8M–O). The above data indicated that silencing CEBPB triggered anti-inflammatory M2-like polarization of macrophages.

### CEBPB Knockdown Weakens Cholesterol Uptake, Esterification and Hydrolysis, and Efflux in ox-LDL–Induced Macrophages

**FIGURE 9:**
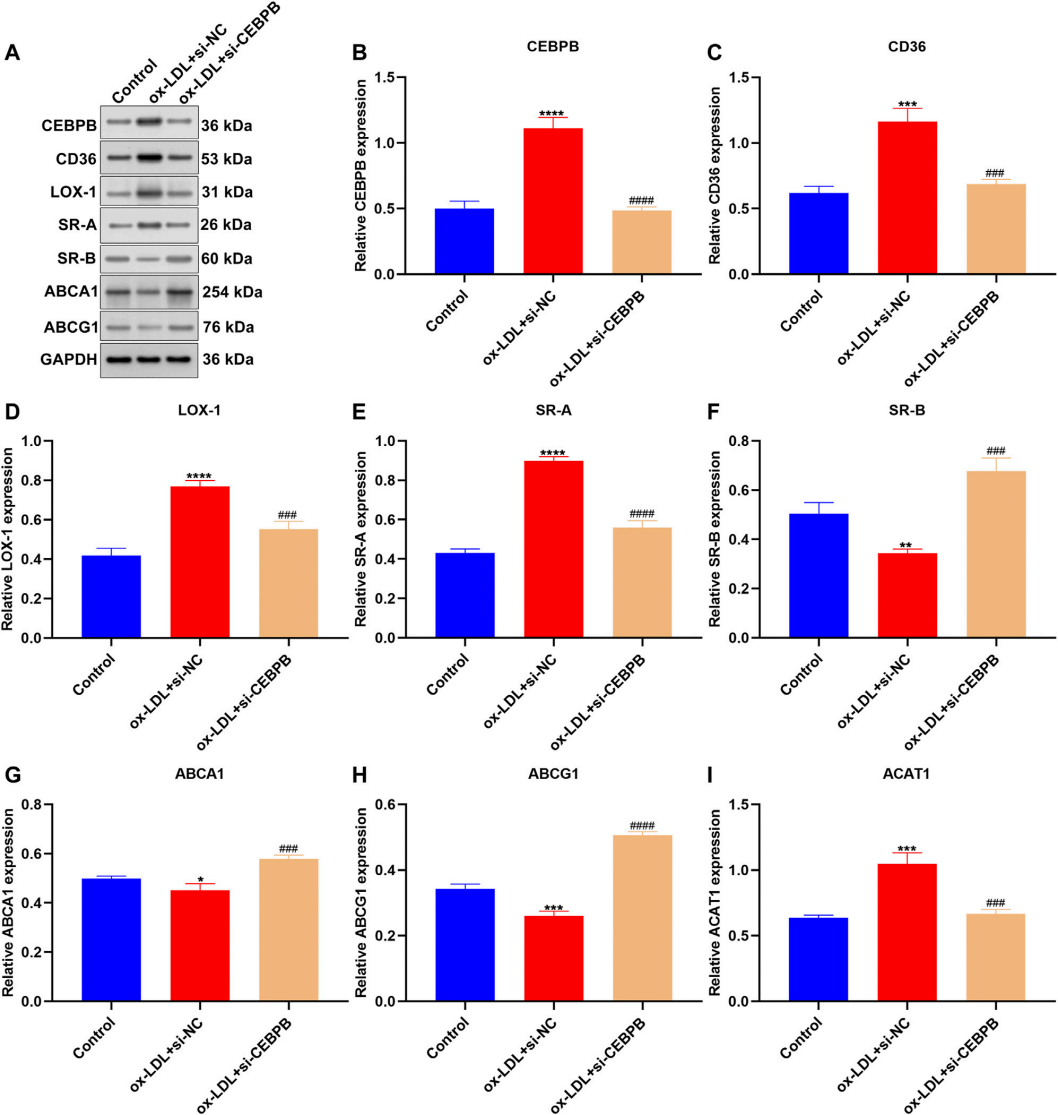
CEBPB knockdown weakens cholesterol uptake, esterification and hydrolysis, and efflux in ox-LDL–induced macrophages. **(A–I)** Western blotting was adopted for detection of the expression of CEBPB, CD36, LOX-1, SR-A, SR-B, ABCA1, ABCG1, and ACAT1 in ox-LDL–induced macrophages with CEBPB knockdown. Compared with control, *p < 0.05; **p < 0.01; ***p < 0.001; ****p < 0.0001. Compared with ox-LDL + si-NC, ^###^ *p* < 0.001; ^####^ *p* < 0.0001.

This study constructed AS cellular models through exposure to ox-LDL in macrophages. Western blotting confirmed the marked up-regulation of CEBPB expression in ox-LDL–induced macrophages (Figures 9A,B). To investigate the effects of CEBPB on AS progression, CEBPB expression was significantly silenced. Cholesterol uptake is a biologic process by which ox-LDL is absorbed through macrophages with an SR-mediated pathway (Wang et al., 2019). Macrophages primarily express a few SR proteins, which bind, internalize, and degrade ox-LDL including CD36, LOX-1, and SR-A. We noticed that CEBPB knockdown weakened the expression of CD36, LOX-1, and SR-A in ox-LDL–exposed macrophages (Figures 9C–E). To maintain intracellular cholesterol homeostasis, excessive cholesterol will be removed from macrophages primarily via transporter-dependent cholesterol efflux pathways such as SR-B, ABCA1, and ABCG1 (Wang et al., 2019). Our results showed the marked increase in SR-B, ABCA1, and ABCG1 expressions in ox-LDL–exposed macrophages after CEBPB was silenced (Figures 9F–H). ACAT1 may re-esterify free cholesterol, thereby blocking the underlying cellular toxicity caused by excessive free cholesterol accumulation. In Figure 9I, CEBPB knockdown reduced the expression of ACAT1 in ox-LDL–induced macrophages. CEBPB knockdown weakened cholesterol uptake, esterification and hydrolysis, and efflux in ox-LDL–induced macrophages.

## Discussion

Aging serves as one of the strongest risk factors of AS (Tyrrell et al., 2020). To decipher the mechanism by which aging triggers AS is of importance for the development of novel therapeutic strategies to decrease the burden of AS due to aging. Herein, this study conducted a comprehensive analysis of the aging-related genes in AS. Our findings showed an aging-related gene signature as a diagnostic marker of AS. ROC curves confirmed the excellent diagnostic capacity of AS. Moreover, we developed two aging-based molecular subtypes across AS samples, with diverse immune infiltrations. Through WGCNA, we identified molecular subtype–derived key genes that were involved in immune activation. *In vitro*, CEBPB knockdown triggered anti-inflammatory M2-like polarization of macrophages as well as weakened cholesterol uptake, esterification and hydrolysis, and efflux in ox-LDL–induced macrophages, suggesting that CEBPB participated in AS progression.

Through differential expression analysis, we identified AS-specific aging-related genes. Our GO and KEGG enrichment analyses uncovered that AS-specific aging-related genes participated in modulating biological processes (oxidative stress, aging, and DNA repair) AS-related (lipid and AS) and immunity-related pathways (TNF and IL-17 signaling pathways), indicative of the prominent biological significance of AS-specific aging-related genes in AS pathogenesis. Through the LASSO method, we developed an aging-based gene signature that was capable of diagnosing AS. AS represents a systemic chronic inflammatory disease involving activated innate immune responses (Gencer et al., 2021). AS lesion is filled with immune cells that may orchestrate and trigger inflammatory response (Bonacina et al., 2021). Increasing evidence suggests the inherent immune diversity in AS plaques (Shen et al., 2021). Moreover, immune cell dysfunction such as aberrant distribution of abundance and type contributes to AS progression (Wang et al., 2021). An in-depth exploration of immune infiltrations in AS and non-AS specimens may offer a better understanding of AS progression. Anti-inflammatory therapy will be considered in the context of other therapies aimed at reducing the harmful biological effects of aging. Herein, according to immune cell infiltrations (central memory CD8 T cells, neutrophils, and CD56bright natural killer cells), mRNA expression of HLAs (HLA-E, HLA-DPB1, and HLA-DRB5), and immune checkpoints (TNFSF14, ICOSLG, and TNFRSF25), there was the prominent heterogeneity in immune infiltrations between AS and non-AS specimens. Moreover, two aging-based molecular subtypes were characterized by diverse immune infiltrations across AS specimens. Among AS-specific aging-related genes, our results confirmed that CEBPB knockdown triggered anti-inflammatory M2-like polarization of macrophages.

Macrophage foam cells act as a key ingredient of atherosclerotic lesions, which exert a critical function in AS progression (Cao et al., 2019). In the early stage, monocytes migrate into the intimal tissues, thereby differentiating into macrophages (Tian et al., 2020). Macrophage phagocytosis and metabolism of ox-LDL are enhanced, and lipid is transported from the cell to the vessel wall. When the uptake of ox-LDL may exceed the metabolic ability of macrophages, macrophages are transformed into foam cells and facilitate AS progression (Peng et al., 2020). Herein, our results showed the up-regulation of CEBPB expression in ox-LDL macrophages. But silencing CEBPB weakened the expression of markers of cholesterol uptake, esterification and hydrolysis, and efflux in ox-LDL macrophages. Thus, CEBPB possessed the potential as a therapeutic target against AS. Nevertheless, several limitations should be pointed out. Firstly, our results were based on analysis of gene expression curated from microarray profiles, but gene expression may not be directly equivalent to protein expression. Secondly, the functions of CEBPB in AS progression will be investigated in AS animal models.

## Conclusion

Collectively, this study constructed an aging-based diagnostic gene signature and two molecular subtypes with diverse immune infiltrations in AS, which suggested the key implications of aging-related genes in diagnosing AS and modulating immune infiltrations.

## Data Availability

The datasets presented in this study can be found in online repositories. The names of the repository/repositories and accession number(s) can be found in the article/Supplementary Material.

## Abbreviations

AUC, area under the ROC curve; AS, atherosclerosis; ELISA, enzyme-linked immunosorbent assay; FDR, false discovery rate; GEO, Gene Expression Omnibus; GO, Gene Ontology; GSEA, Gene Set Enrichment Analysis; GSVA, Gene Set Variation Analysis; HAGR, Human Ageing Genomic Resources; HLA, human leukocyte antigen; IL-6, interleukin-6; KEGG, Kyoto Encyclopedia of Genes and Genomes; LASSO, least absolute shrinkage and selection operator; MCODE, Molecular Complex Detection; PPI, protein–protein interaction; ROC, receiver-operator characteristic; RT-qPCR, reverse transcription-quantitative polymerase chain reaction; STRING, Search Tool for the Retrieval of Interacting Genes/Proteins; ssGSEA, single sample gene set enrichment analysis; TNF-α, tumor necrosis factor-α; WGCNA, weighted gene correlation network analysis.
